# Periodontal Disease and Tooth Wear in a Sample of German Soldiers with Posttraumatic Stress Disorder

**DOI:** 10.3290/j.ohpd.b1993989

**Published:** 2021-09-11

**Authors:** Felix Wörner, Thomas Eger, Ursula Simon, Anne Wolowski

**Affiliations:** a Dentist and Statistician, Bundeswehr Central Hospital Koblenz, Department of Dentistry-Periodontology, Koblenz, Germany. Methodology and investigation, software, data curation, prepared original draft, read and agreed to the published version of the manuscript.; b Periodontist, Clinical Director, Bundeswehr Central Hospital Koblenz, Department of Dentistry-Periodontology, Koblenz, Germany. Conceptualisation, methodology and investigation, writing and review, project administration and funding acquisition, read and agreed to the published version of the manuscript.; c Psychiatrist, Clinical Director, Bundeswehr Central Hospital Koblenz, Department and Center for Mental Health and Psychiatry, Koblenz, Germany. Project administration and funding acquisition, proofread the manuscript, contributed substantially to discussion, read and agreed to the published version of the manuscript.; d Prosthodontist, University Hospital and Faculty of Medicine Münster, Department of Prosthodontics and Biomaterials, Münster, Germany. Conceptualisation, hypotheses, proofread the manuscript, read and agreed to the published version of the manuscript.

**Keywords:** attrition, bruxism, periodontitis, PTSD, tobacco

## Abstract

**Purpose:**

Dental symptoms of post-traumatic stress disorder (PTSD) patients include a majority of painful temporomandibular joint and masticatory muscle findings, restricted mouth opening, and pronounced attritions. Traumatic occlusal force resulting in injury of the teeth and/or the periodontal attachment apparatus may exceed the adaptive capacity of the individual person or site. This observational cross-sectional study in soldiers with PTSD and a non-PTSD control group after military deployments aimed to evaluate a possible relationship between bruxism and periodontal diagnosis.

**Materials and Methods:**

Ninety-six in-patients and 27 out-patients (21 women, 102 men) with specialist-confirmed PTSD and bruxism after up to 17 foreign assignments, and 36 male non-PTSD controls with up to 15 foreign assignments underwent general dental, functional, and periodontal examinations.

**Results:**

All three groups showed no statistically significant differences in terms of age (34.8 ± 8.6 years), number of teeth (n: 26.3 ± 3.4), status of dentition (DMFT 9.7 ± 6.6), incidence of periodontitis (36%) and recessions (n: 5.8 ± 5.7). From the control group to the out-patient group to the in-patient group, the proportion of smokers and tobacco use increased statistically significantly, as did the extent of attrition. In the in-patient group, with statistically significantly lower educational levels, the number of perceived prophylaxis sessions was statistically significantly reduced in the last two years.

**Conclusions:**

Taking into account the retrospective recording of the last traumatic event, the average time of five years until therapy does not seem to have any consequences for the frequency and severity of inflammatory periodontal disease, recession, and wedge-shaped defects in soldiers with bruxism in PTSD, regardless of the need for in-patient or out-patient treatment.

The influence of psychosomatic factors on the development of periodontitis was first described by Miller and Firestone in 1947.^33^ Periodontal disease is a pandemically non-communicable disorder^21,46^ with serious socioeconomic consequences and a high burden on the quality of life.^8,44,47^ It is a chronic multifactorial inflammatory disease associated with dysbiotic dental plaque biofilms.^40^ Periodontitis is characterized by the progressive destruction of the tooth-supporting apparatus.^37^ If untreated, it may lead to tooth loss, although it is preventable and treatable in the majority of cases.^37^ A variety of systemic diseases and conditions can affect the course of periodontitis or have a negative impact on the periodontal attachment apparatus. Traumatic occlusal force is defined as any occlusal force resulting in injury of the teeth and/or the periodontal attachment apparatus. These were historically defined as excessive forces to denote that the forces exceed the adaptive capacity of the individual person or site. The presence of traumatic occlusal forces may be indicated by one or more of the following: fremitus, tooth mobility, thermal sensitivity, excessive occlusal wear, tooth migration, discomfort/pain on chewing, fractured teeth, radiographically widened periodontal ligament space, root resorption, and hypercementosis.^19^ Evidence from animal models indicate that traumatic occlusal forces may increase alveolar bone loss.^20,51^ There is evidence from observational studies that traumatic occlusal forces may be associated with the severity of periodontitis.^18^

Bruxism is a multifaceted phenomenon that has been associated with several factors mediated by the central nervous system. According to an updated international consensus in 2018, bruxism is a repetitive masticatory muscle activity that is not necessarily a disorder in healthy individuals.^25^ The prevalence of bruxism in the general population is approximately 25%,^30^ whereas a two-fold rate of bruxism (53%) has been reported in periodontal patients.^31^ Therefore, the role of bruxism in periodontal disease merits further research. Bruxism effects and associated factors on stomatognathic structures are considerably heterogeneous and inconsistent. Factors consistently associated with bruxism are use of alcohol, caffeine, tobacco, some psychotropic medications, esophageal acidification, and second-hand smoke. Furthermore, temporomandibular disorder (TMD) signs and symptoms present a plausible association.^32^ Current knowledge is mostly related to sleep bruxism.

Posttraumatic stress disorder (PTSD) is a disorder that may develop following exposure to an extremely threatening or horrific event or series of events.^41^ It is characterised by all of the following: 1) re-experiencing the traumatic event or events in the present in the form of vivid intrusive memories, flashbacks, or nightmares. These are typically accompanied by strong or overwhelming emotions, particularly fear or horror, and strong physical sensations; 2) avoidance of thoughts and memories of the event or events, or avoidance of activities, situations, or people reminiscent of the event or events; and 3) persistent perceptions of heightened current threat, for example as indicated by hypervigilance or an enhanced startle reaction to stimuli such as unexpected noises. The symptoms persist for at least several weeks and cause significant impairment in personal, family, social, educational, occupational or other important areas of life.^7,17,41^ Epidemiological estimates of the prevalence of the various trauma sequelae disorders vary considerably depending on the sample and time point studied, among other factors.^41^ Kindler et al^23^ estimated the association between signs of TMD and symptoms of PTSD in a representative sample from the general population of north-eastern Germany. Subjects having clinical PTSD (n = 62) had a 2.56-fold increase in joint pain and a 3.86-fold increase in muscle pain compared to subjects without clinical PTSD.

In many cases, the first psychiatric evaluation of affected patients does not occur until years after the traumas.^7,17,41^ Dental problems in soldiers with PTSD include blood and injury phobia, dental anxiety, tooth grinding, restricted mouth opening, painful TMJ clicking, dentin hypersensitivity, and marked tobacco use.^7^

Possible correlations of periodontal diagnosis, adherence to professional prophyaxis and education about excessive occlusal force, tooth wear, age and smoking habits for PTSD in- or out-patients vs non-PTSD controls with the same number of military deployments have not been analysed until now. The aim of the present study was to evaluate a possible relationship between different stadiums of inflammatory periodontal disease, attritions and non-carious cervical lesions in soldiers with either in- or out-patient treatment for bruxism and PTSD.

## Materials and Methods

All soldiers who had received at least three months of psychiatric and dental treatment for craniomandibular disease and bruxism were recruited. Out-patients lived close to the hospital with their families. Subjective distress caused by traumatic events was measured by the Impact of Event Scale – Revised (IES-R).^28^ Items correspond directly to 14 of the 17 DSM-IV symptoms of PTSD. Respondents were asked to identify a specific stressful life event and then indicate how much they were distressed or bothered during the past seven days by each “difficulty” listed. The IES-R yields a total score (ranging from 0 to 88); subscale scores can also be calculated for the Intrusion, Avoidance, and Hyperarousal subscales. Soldiers with a minimum total score of 35 were included. The IES-R is not intended to be used as a proxy for a diagnosis of PTSD but rather allows an estimation of symptom severity.^28^

All patients were examined by a periodontist between August 2014 and December 2019 at the Bundeswehr Central Hospital in Koblenz, Germany. Exclusion criteria were pregnancy, known former use of narcotic substances and acid-reflux–induced erosions.

Clinical dental examination for decayed missing and filled teeth, full-mouth periodontal status with six sites per tooth for probing depth and recessions and functional analysis of the temporomandibular joint were performed. The periodontal diagnosis was made according to the classification scheme defined in the 2017 World Workshop on the Classification of Periodontal and Peri-Implant Diseases and Conditions.^48^ Examining a new patient consisted of 4 sequential steps:^37^ identifying a patient suspected of having periodontitis, confirming the diagnosis of periodontitis, staging the periodontitis case, and grading the periodontitis case. For full-mouth periodontal probing, clinical attachment loss and bleeding on probing measurements at six sites per tooth, a pressure-calibrated manual periodontal probe was used (Aesculap DB764R; Tuttlingen, Germany). Self-administered questionnaires were used for smoking habits, medical, dental and social history. Anamnesis for bruxism was followed by a personal interview with 5 questions:^38^

- Has anyone heard you grinding, clenching or chattering with your teeth at night (every night/day during the last 5 days)?
- Do your jaws feel stiff or fatigued when you wake up in the morning?
- Do you have headache in the area of your temples?
- Do you find yourself grinding your teeth during daytime?
- Do you find yourself clenching your teeth during daytime?

Differentiation between waking and sleeping bruxism was made via interview. For the quantification of non-carious cervical lesions (NCCL), the definitions of Allen and Winter for five grades (0-4) were accepted.^2^ The presence of bruxism was diagnosed clinically by muscle palpation according to the guideline on RDC-TMD. This was done solely by one trained dentist, in order to minimise variance. For quantification of attrition a modified Tooth Wear Index (mTWI)^50^ on casts was used (Table 1). Here, the mean mTWI was calculated for each patient from the attritions of all measurable non-overcrowned teeth. Comparable data for crown length and width on 17- to 21-year-old males by Björndal et al^4^ were used for calibration. After oral hygiene instruction and plaque disclosure, all patients and subjects of the study revceived professional tooth cleaning or sufficient non-surgical periodontal therapy. In cases of functional TMJ disease with pain and bruxism (123 PTSD patients and 1 control-group subject), conservative treatment of occlusal dysfunctions with an open-bite aid for nocturnal use (modified acrylic interceptor) was performed.^42^

**Table 1:** Description of the ordinal grading scales for occlusal/incisal tooth wear

Attrition grading scale (Wetselaar et al^50^)	Description	Modified study code (mTWI)
0	No wear	0
1a	Initial, minimal wear within the enamel cusps or incisal tips	1
1b	Facets within the enamel parallel to the normal planes of contour	2
1c	Noticeable flattening of cusps or incisal edges within the enamel	3
2	Wear with dentin exposure and loss of clinical crown height of < 1/3	4
3a	Wear with dentin exposure and loss of clinical crown height of 1/3 – 1/2	5
3b	Wear with dentin exposure and loss of clinical crown height of 1/2 – 2/3	6
4	Wear with dentin exposure and loss of clinical crown height of > 2/3	7


The non-control group was matched for ages of 30 to 45 years, number of teeth ≥ 24 and at least two military deployments during the last 5 years.

Descriptive data are presented as the means, standard deviation or n and percentage. The statistical analysis was performed using SPSS 24 (IBM; Armonk, NY, USA). The Kruskal-Wallis test and Bonferroni’s correction were used to test for group differences in mTWI. Possible confounding variables, such as stages of periodontitis, location, number of teeth, smoking as a risk factor, and gender were considered. Normal distribution was tested with the Kolmogorov-Smirnov and Shapiro-Wilk tests. Non-parametric procedures were used. Spearman’s correlation was also calculated.

The statistical significance level was set at p < 0.05. Sample size was based on the total number of in-patient or out-patients referred by the Department of Mental Health for splint therapy because of bruxism with a more than 5-day period of tooth grinding from August 2014 to December 2019. All patients had to be treated in-patient or out-patient for a time period of more than 6 weeks.

### Ethics Statement

The present investigation was conducted by clinical questionnaires, examinations and multiple patient-dentist talks in the Department for Dentistry-Periodontology, Periodontology of the Bundeswehr Central Hospital Koblenz. This cross-sectional study was conducted at the Bundeswehr Medical Service Academy (Munich, Germany) and registered in the military clinical trial register (12K1-S-80 1414). In full accordance with ethical principles, the guidelines of the Helsinki Declaration were followed and the Regional Ethics Review of the State Physicians Association of Rhineland-Palatinate in Germany (837.068.14/9307-F) approved the study (28 March 2014). Subjects were informed that they could cease participation in the study at any time without any consequence. All participants were military personnel. Written informed consent was obtained from all subjects involved in the study after written information of all patients referred to us. Subjects were informed about their voluntary inclusion and that they could leave the study at any time without any consequence.

## Results

All patients were in-patient (n: 96) or out-patient (n: 27), and referred by the Department of Mental Health for treatment of bruxism. Before participation, all 123 PTSD patients and 36 non-PTSD subjects of the same age group with multiple military deployments gave written informed consent to their examination. Patients (21 females, 102 males) had a history of up to 17 military deployments, and 36 out-patient male non-PTSD controls with up to 15 military deployments were examined in terms of general dentistry, function and periodontology. The average time between the last traumatic event during military deployment abroad and the first psychiatric presentation at the hospital for the 123 PTSD patients was 4.65 ± 3.12 years. IES-R total scores for in-patient PTSD and out-patient PTSD patients showed no statistically significant differences (46,7 ± 9.4 vs 47.0 ± 11.3).

All three groups showed no statistically significant differences in age (34.8 ± 8.6 years), number of teeth (n: 26.3 ± 3.4), dental care status (DMFT 9.7 ± 6.6), incidence of periodontitis (36%), and recessions (n: 5.8 ± 5.7) (Table 2). Ranging from the non-PTSD control group to the out-patient group to the in-patient PTSD group, the proportion of smokers and other forms of tobacco use increased statistically significantly, as did the extent of functional impairment and attrition. In the in-patient group, with significantly lower levels of education, the number of prophylaxis sessions was statistically significantly reduced in the last two years. Normal distribution was tested with the Kolmogorov-Smirnov and Shapiro-Wilk test. This was rejected for most variables. Therefore, non-parametric procedures were used.

**Table 2:** Baseline anamnestic and clinical data in PTSD in-patient, PTSD out-patient and non-PTSD control group (n: 159)

	PTSD in-patient	PTSD out-patient	Non-PTSD control group
Number	96	27	36
Men/women	86/10	16/11	36/0
Age (years)	34.6 ± 8.3	37.2 ± 11.5	34.4 ± 4.2
Number of assignments abroad	2.8 ± 3.4	1 ± 1.17	6.0 ± 4.6
Smoking percentage	67%*	44%*	28%*
Number of teeth	26.3 ± 2.7	26.3 ± 2.3	27.1 ± 11.1
DMFT	9.8 ± 7.0	9.8 ± 7.0	9.3 ± 6.0
Prophylaxis sessions in the last 2 years	0.8 ± 0.9*	1.1 ± 1.2*	1.9 ± 0.3*
Educational degree: (n) degree	6	7	15
12 years	16	6	9
10 years	45	11	6
9 years	39	3	6
mTWI	4.0 ± 0.7*	3.0 ± 0.9*	1.3 ± 0.8*

*The groups differ significantly from each other with regard to the smoking percentage (Kruskal-Wallis, chi-squared 17.01), prophylaxis sessions (Kruskal-Wallis p < 0.01, chi-squared 37.83) and mTWI (Kruskal-Wallis p < 0.01, chi-squared 92.14).

**Fig 1:**
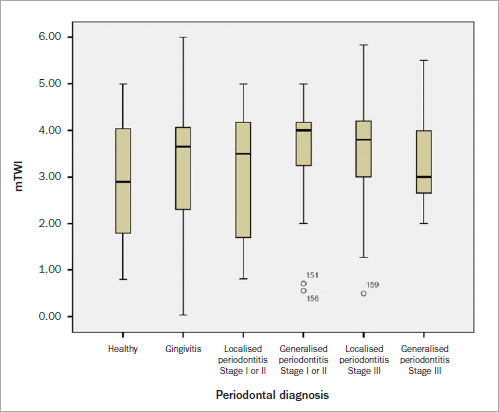
Correlation between periodontal diagnosis and modified tooth wear index (mTWI) (n:159).

The groups differed statistically significantly (p < 0.01) from each other with regard to the attrition index (mTWI) (Dunn-Bonferroni test, Kruskal-Wallis test, mTWI not normally distributed). There was no correlation between attritions, cervical wedge-shaped tooth defects, and recessions. Periodontal diagnosis did not correlate with mTWI (rs = 0.078, p = 0.329 n =159) (Fig 1). Periodontal diagnosis did not correlate with group membership (rs = -0.039, p = 0.622, n =159) (Table 3). Group membership correlates with prophylaxis sessions (rs = 0.363, p < 0.01, n = 159). There was no correlation between prophylaxis sessions and periodontal diagnosis (rs = 0.07, p = 0.379, n = 159) and no correlation between periodontal diagnosis and education. Periodontal diagnosis correlated with daily tobacco use of > 10 cigarettes/day (rs = 0.319, p < 0.01, n = 159) (Table 4) and subject’s age (rs = 0.322, p < 0.01, n = 159). These were medium effects according to Cohen (Table 5). Education and PTSD were related (chi-squared = 33.5, p < 0.01, n = 159, Cramer’s V = 0.325).

**Table 3:** Correlation between periodontal diagnosis and group membership (PTSD-In-patient, PTSD-out-patient, non-PTSD control group)

	n:	Periodontally healthy	Gingivitis	Localised periodontitis Stage I or II	Generalised periodontitis Stage I or II	Localised periodontitis Stage III	Generalised periodontitis Stage III	Periodontitis Stage I-III (%)
PTSD out-patient	27	9	7	1	4	0	6	41
PTSD in-patient	96	7	52	13	9	10	5	39
Control group	36	4	21	7	2	2	0	30
Total n	159	20	80	21	15	12	11	

Periodontal diagnosis did not correlate with group membership (rs = -0.039, p = 0.622, n =159).

**Table 4:** Correlation between smoking status and periodontal diagnosis (n: 159)

	Periodontal diagnosis						Total
	Healthy	Gingivitis	Localised Stage I or II	Generalised Stage I or II	Localised Stage III	Generalised Stage III	
Non-smoker	16	40	7	4	4	2	73
Smoker	4	40	14	11	8	9	86
Total	20	80	21	15	12	11	159

Periodontal diagnosis correlated with daily tobacco use of > 10 cigarettes/day (rs = 0.319, p < 0.01, n = 159).

**Table 5:** Correlation between age and periodontal diagnosis (n: 159)

	Periodontal diagnosis					
	Healthy	Gingivitis	Localised Stage I or II	Generalised Stage I or II	Localised Stage III	Generalised Stage III
Age	30.6 ± 4.8	34.3 ± 7.4	33.1 ± 6.7	39.5 ± 8.5	36.8 ± 8.1	45.6 ± 11.3

Periodontal diagnosis correlated with age (rs = 0.322, p < 0.01, n = 159).

The data presented in this study are also available on request from the corresponding author.

## Discussion

The incidence of severe periodontal disease (stage III) among soldiers with PTSD was 17%, significantly higher than that in the German population at 8.2% according to the DMS V study^43^ and that in the non-PTSD control group in our study at 5.5%. Our results confirm the negative relationship of education with moderate to severe periodontitis.^5^

There is a well-established negative association between educational level, as well as other traits related to cognitive ability, and PTSD and economic status as a mediator.^39^ In our cross-sectional study, various risk factors were presented, such as the complaint-oriented utilisation of dental therapy, the high proportion of smokers, and the educational status/social status of the PTSD group.

It is well established that smoking has a major adverse effect on the periodontal supporting tissues, increasing the risk of periodontitis by 2- to 5-fold.^49^ Tobacco smoking is a prevalent behaviour with severe health consequences. Although tobacco use was once classified as a habit, it is now considered a nicotine dependence and a chronic relapsing medical disorder.^17^ The severity of periodontal diagnosis in our study correlates with smoking status (> 10 cigarettes/day). The proportion of smokers among PTSD in-patients in our study is disproportionately high.

Periodontal diagnosis in our study of soldiers with PTSD did not correlate with the severity of attrition (mTWI). In recent studies of patients with deployment-related PTSD from the United States^14^ and Israel,^45^ the majority of soldiers were found to have craniomandibular dysfunction and inflammatory periodontal disease of varying severity. The effect of tobacco and alcohol use, as well as psychopharmacological therapy on these patients in terms of the development of periodontal disease and craniomandibular dysfunction has not yet been determined.

There is limited evidence from human and animal studies that traumatic occlusal forces can cause inflammation in the periodontal ligament.^13^ Only two studies have addressed the association between bruxism and tooth loss due to periodontal disease (TLPD) in patients following periodontal maintenance therapy.^31,36^ The results of these studies were consistent and showed that bruxism doubled the risk of TLPD, which is similar to smoking. The combination of heavy smoking and bruxism increased the number of teeth lost.^31^

However, bruxism is poorly understood and represents one of the most controversial issues in dentistry. Consequently, it is a matter that deserves serious scientific discussion.^25^ Botelho et al^6^ analysed 1064 participants (427 non-periodontitis and 637 with periodontitis) recruited during an epidemiologic study carried out in the southern region of the Lisbon Metropolitan Area, Portugal. In that study, bruxers were less likely to develop periodontitis and had better periodontal clinical characteristics. Nakayama et al^35^ analysed 57 patients with periodontal disease. No statistically significant difference was found in the comparison of maximum community periodontal index proportions between individuals with high sleep-bruxism–related signs and high waking-bruxism–related signs. Sleep bruxism has attracted attention as a factor influencing periodontal disease, and their data suggest that patients with periodontal disease demonstrate more bruxism while awake than while asleep.^35^

Kato et al^22^ took continuous recordings of sixteen subjects with no or mild periodontitis and fifteen subjects with moderate or severe periodontitis with surface electromyography of the masseter muscles. The results of that study suggested that masseter muscle activity might be related to the severity of periodontitis.

Bruxism is not currently considered to have a causal cure.^25^ Bruxism can be associated with substantial non-carious tooth structure loss and/or loss of restorative materials and poses a risk for technical and biological failure of dental restorations.^26^ Studies show a higher prevalence of craniomandibular dysfunction (CMD) symptoms, such as pain in the masticatory muscles or temporomandibular joints, headache, and muscle tension, in patients with bruxism.^1^

The aetiology of bruxism is multifactorial and partly unknown.^25,26,29,30.34^ Results from longitudinal studies identifying risk factors are lacking. Peripheral factors, which include dental occlusion or morphological characteristics of cranial or jaw growth, are currently considered to be more secondary to bruxism,^26^ or it is concluded that there is no evidence that occlusion and bruxism are aetiologically related.^25^ Waking bruxism appears to be more psychologically based (emotional stress and other emotional factors),^12^ while sleep bruxism is considered more of a central nervous disorder.^29^

There is no evidence that traumatic occlusal forces lead to periodontal attachment loss, non-carious cervical lesions, or gingival recessions.^19^ We also did not find that traumatic occlusal forces in PTSD patients caused non-carious cervical lesions. Abfraction, a term used to define a wedge-shaped defect that occurs at the cementoenamel junction of affected teeth, has been claimed to be the result of flexure and fatigue of enamel and dentin. There is also evidence from observational studies that occlusal forces do not cause gingival recession.^3,15^ A relationship between attritions, bruxism and PTSD has been demonstrated.

These results should encourage general practitioners and dentists to acknowledge the role of PTSD and traumatic events in the diagnosis and therapy of TMD, especially in a period of international migration and military assignments abroad.^23^

Bruxism in patients with PTSD and periodontal disease are now a dental challenge. Periodontal disease can reduce the oral-health–related quality of life, but its treatment can lead to improvements. During periodontal treatment, patients must know and comprehend what periodontal disease is and why adhering to the therapeutic program is key to initial and long-term treatment success.^11,27^ It is necessary to presentat personalised psychosocial benefits of periodontal therapy for treatment success at all stages of therapy.^16^

Depression is a risk factor for noncompliance in all somatic diseases,^10^ and leads to an altered immune response in periodontitis.^24^ Nonsurgical periodontal therapy significantly reduces several stress hormones at the local level.^9^

### Limitations of the Study

The study included only soldiers as patients. Soldiers undergo mandatory dental examinations by the military to determine their dental fitness. Dental treatment, on the other hand, is mandatory only for deployment. Soldiers are at higher risk for the development of PTSD after military deployment all over the world.^7^ PTSD prevention efforts are therefore still needed. Although the sample size was small, 123 of 128 soldiers with bruxism referred between 2014 and 2019 to the Department of Dentistry-Periodontology from the Bundeswehr Central Hospital Koblenz, Department and Center for Mental Health and Psychiatry were recruited. Military dentists’ empathy towards their patients’ stress reaction to periodontal treatment needs for military deployments have not been analysed yet. It has not yet been clarified whether burnout or other mental disorders after military deployments could be prevented by an early determination of bruxism associated attritions and, if necessary, resilience training or early intervention in the case of the latter.^7,41^

## Conclusions

Dental care of PTSD patients prior to in-patient psychiatric therapy was characterised by fewer visits for dental prophylaxis and high tobacco use.

Taking into account the retrospective documentation of the last traumatic event, the average time of five years until therapy does not seem to have any consequences for the incidence of inflammatory periodontal disease, recession, and wedge-shaped defects in soldiers with PTSD, regardless of the need for in-patient or out-patient treatment. A tendency toward periodontitis with higher stages and grades exists in PTSD patients. Bruxism has no effect on periodontitis.

The extent of bruxism-related attritions as well as the more frequently omitted dental preventive measures due to phobias developed due to the disease in PTSD patients may lead to significant dental treatment needs. Clinical management of traumatic occlusal forces is indicated to prevent and treat these signs and symptoms.
